# Improved Thermoelectric Performances of SrTiO_3_ Ceramic Doped with Nb by Surface Modification of Nanosized Titania

**DOI:** 10.1186/s11671-016-1407-8

**Published:** 2016-04-12

**Authors:** Enzhu Li, Ning Wang, Hongcai He, Haijun Chen

**Affiliations:** State Key Laboratory of Electronic Thin Films & Integrated Devices, University of Electronic Science and Technology of China, Chengdu, 610054 People’s Republic of China

**Keywords:** Nb-doped SrTiO_3_, Surface modification, Nanosized titania, Thermoelectric performance

## Abstract

Nb-doped SrTiO_3_ ceramics doped with the surface modification of nanosized titania was prepared via liquid phase deposition approach and subsequent sintered in an Ar atmosphere. The surface modification of nanosized titania significantly improved the ratio of the electrical conductivity to thermal conductivity of SrTiO_3_ ceramic doped with Nb, and has little impact on the Seebeck coefficient, thus obviously improving the dimensionless thermoelectric figure of merit (*ZT* value). The surface modification of nanosized titania is a much better method to lower the thermal conductivity and to enhance the electrical conductivity than the mechanical mixing process of nanosized titania. The highest *ZT* value of 0.33 at 900 K was obtained. The reason for the improved thermoelectric performances by the surface modification of nano-sized titania was preliminary investigated.

## Background

More and more attentions have been paid to the bulk nanostructured thermoelectric materials due to their high thermoelectric performances [1–5]. Tang et al. reported the layer nanostructured Bi_2_Te_3_ bulk materials sintered by spark plasma sintering and combined melt spinning technique, and obtained the highest *ZT* value of 1.35 at 300 K [6]. Mi et al. reported that the hot pressed mixture of nanoscale and microsized CoSb_3_ powders formed the n-type CoSb_3_ nanocomposites, and reached the highest *ZT* value 0.71 at 700 K [7]. Poudel et al. prepared nanostructured bismuth antimony telluride alloys via ball milling and hot pressing technique, and the highest *ZT* value reached 1.4 at 373 K [8]. Han et al. reported Yb_0.2_Co_4_Sb_12+*y*_ nanostructured bulk materials by combining melt spinning method with spark plasma sintering, and obtained the highest *ZT* value of 1.26 at 800 K [9]. Han et al. also fabricated *n*-type skutterudites In_*x*_Ce_*y*_Co_4_Sb_12_ with in situ forming nanostructured InSb phase via a melt-quench-anneal-spark plasma sintering technique, and obtained the highest *ZT* value of 1.43 at 800 K [10]. Kadel et al. reported Bi_2_Se_3_ nanostructures by solvothermal method and the highest *ZT* value obtained 0.096 at 523 K [11].

Recently, SrTiO_3_ thermoelectric materials with non-toxic and element-rich advantages arose wide attention [12, 13]. In our previous study, Nb-doped SrTiO_3_ (Nb-STO) with titanate nanotube additions fabricated via the pressure-less sintering method [14–16]. The additions were directly mixed with strontium titanate powders, which induced the inhomogenous distribution of additions and further lead to the inhomogenous thermoelectric performance of bulk ceramic. Thus, to obtain a homogenous thermoelectric performance, it is important to make a homogenous distribution of the additions.

In this study, liquid phase deposition approach was used to surface-modify Nb-STO by forming the nanosized titania on the strontium titanate grains, and its effect on the thermoelectric performances of SrTiO_3_ ceramic doped with Nb were investigated in detail.

## Methods

SrCO_3_, TiO_2_, and Nb_2_O_5_ powders with high purity were used to prepare the single-phase Nb-doped SrTiO_3_ (Sr(Ti_0.85_Nb_0.15_)O_3_, Nb-STO) powders in an Ar atmosphere at 1400 °C for 4 h via a solid-state reaction. As-prepared Nb-STO powders were put into the aqueous solution including (NH_4_)_2_TiF_6_ (0.06 M) and H_3_BO_3_ (0.2 M). The slurry was strongly stirred at room temperature for 2 h. The centrifugated powders were washed several times with de-ionized water and then dried at about 80 °C. The Nb-STO powders without and with surface modification of nanosized titania were then pressed into pellets under a pressure of 20 MPa and sintered in an Ar atmosphere at 1500 °C for 3 h in a graphite crucible.

The microstructure observations were conducted using scanning electron microscopy (SEM, S-3000N, Hitachi Corporation). The thermoelectric performances, the Seebeck coefficient and electrical conductivity, were determined at 300–1000 K in an Ar atmosphere using an automatic thermoelectric measuring apparatus (RZ-2001K, Ozawa Scientific Corporation). The thermal conductivity (*κ*) was determined from the thermal diffusivity (*β*), specific heat capacity (*C*_p_), and density (ρ) using the following equation: *κ* = *ρC*_*p*_*β*. The thermal diffusivity was determined by the common laser flash method (TC-9000V, ULVAC-RIKO Corporation). And the specific heat capacity was determined by a differential scanning calorimeter system (DSC-2910, TA Instruments Corporation).

## Results and Discussion

**Fig. 1:**
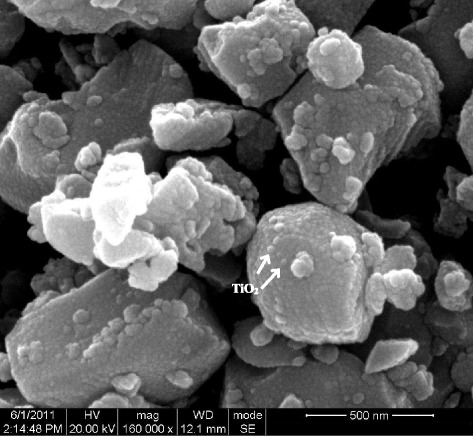
SEM micrograph of raw Nb-STO powders with coated nano-sized titania

**Fig. 2:**
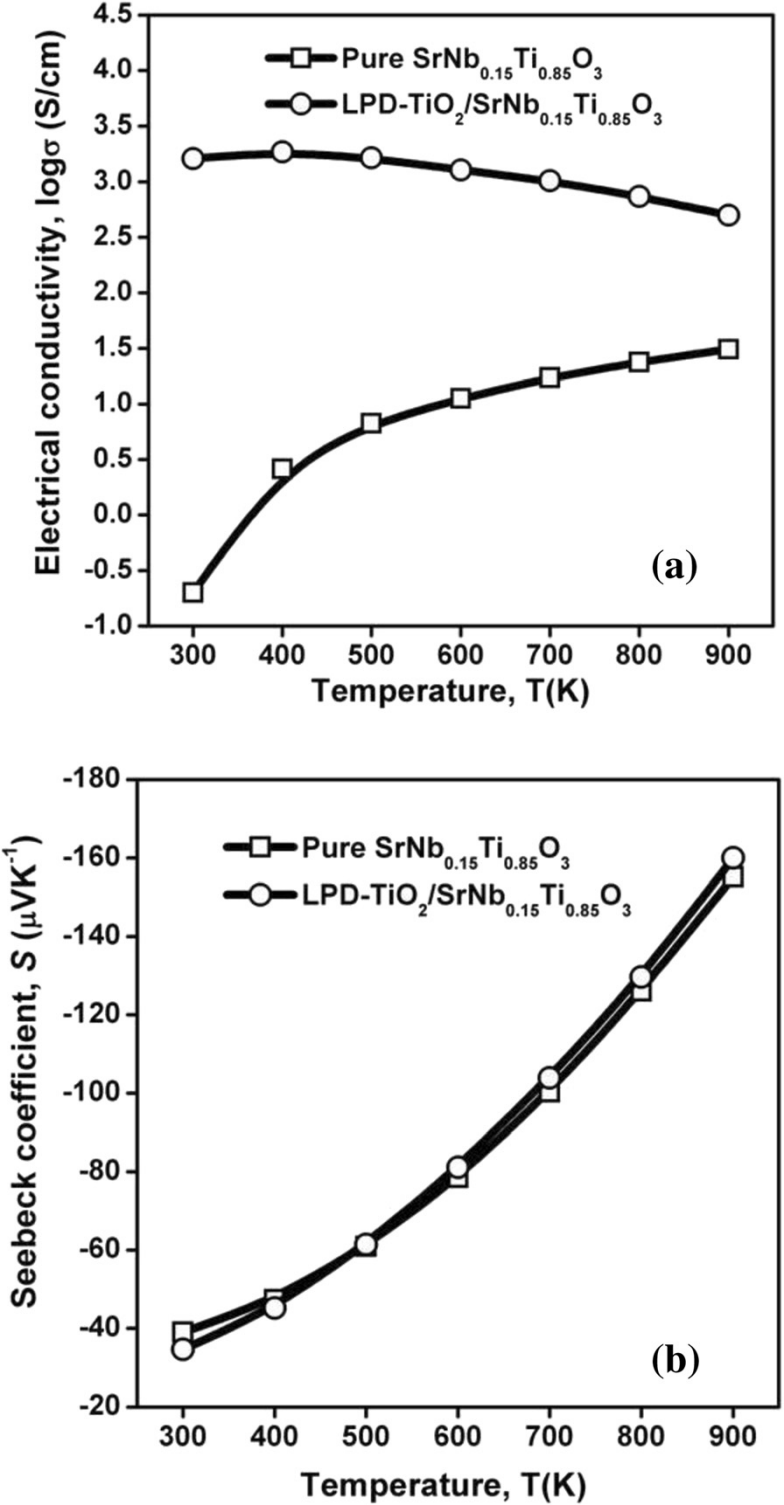
Temperature-dependent electric transport performances of Nb-STO composites with and without the surface modification of nanosized titania: **a** electrical conductivity, **b** Seebeck coefficient

Figure 1 shows the SEM results of raw Nb-STO powders coated with nano-sized titania via liquid phase deposition. The nanosized titania prepared by liquid phase deposition, with anatase phase [17], homogeneously coated on the surface of the Nb-STO powders. Figure 2 shows the relationship between the electrical conductivity (σ)/Seebeck coefficient (*S*) performances and temperature of Nb-STO composites modified by nanosized titania. The results show that the electrical conductivity significantly increased by surface modification of nanosized titania (Fig. 2a). Meanwhile, it has small impacts on the Seebeck coefficient (Fig. 2b).

**Fig. 3:**
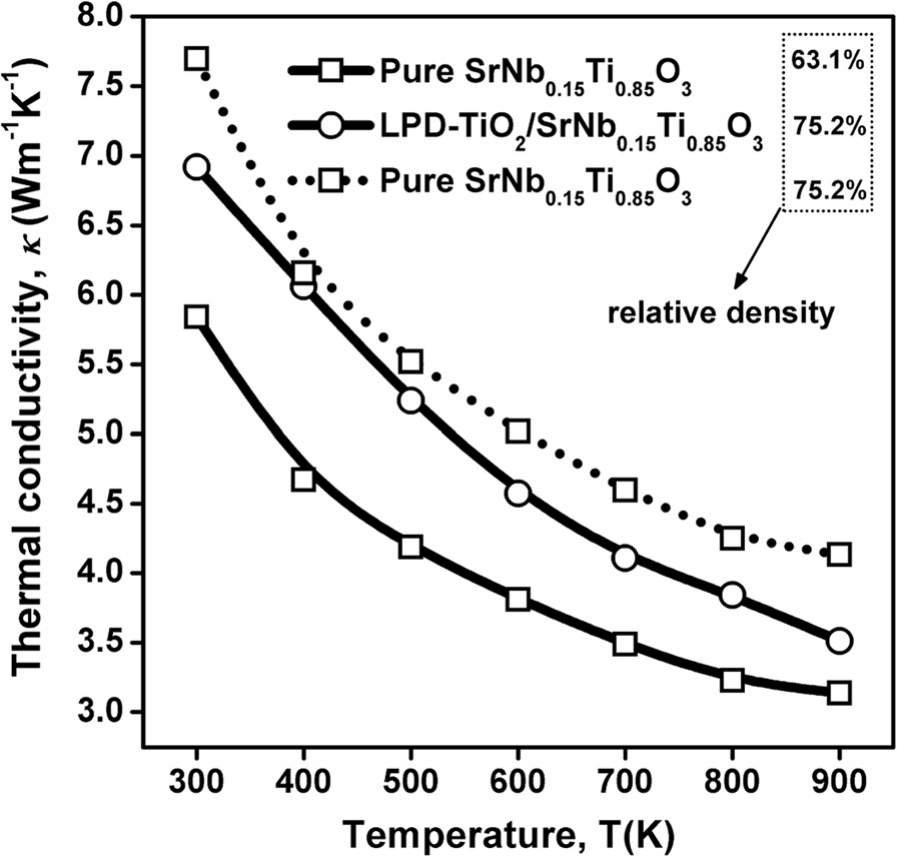
Temperature-dependent measured (*solid line*) and calculated (*dotted line*) thermal conductivities of Nb-STO composites with and without the surface modification of nanosized titania

Figure 3 shows the relationship between the temperature and the measured thermal conductivity of Nb-STO samples with and without surface modification of nanosized titania. Although the results showed that the thermal conductivity enhanced by surface modification of nanosized titania (solid line), the relative densities of the two samples are different: the pure Nb-STO is 63.1 %, and 75.2 % with titania modified, as listed in Fig. 3. Hence, the measured thermal conductivity cannot accurately show the effect of surface modification. To eliminate the effect of porosity, the relative densities of Nb-STO samples with and without surface modification of nanosized titania are assumed to be the same, and the thermal conductivity of the was determined using Klemens’ equation [18]:

1 $$ {\kappa}_1/{\kappa}_0=1-4{\varphi}_1/3, $$

2 $$ {\kappa}_2/{\kappa}_0=1-4{\varphi}_2/3, $$

where *κ*_1_ and *κ*_2_ are the thermal conductivities of the samples with different porosities (φ_*1*□_ and φ_2_)□, and *κ*_0_ is the thermal conductivity of the fully dense sample. From Eqs. (1) and (2), the following relationship could be obtained:

3 $$ {\kappa}_1/{\kappa}_2=\left(1-4{\varphi}_1/3\right)/\left(1-4{\varphi}_2/3\right) $$

Based on Eq. (3), the thermal conductivity of Nb-STO sample without surface modification, which has the same relative density to the sample with surface modification of nanosized titania, 75.2 %, was obtained, shown by dotted lines in Fig. 3. The results indicate that the surface modification of nanosized titania could actually reduce the thermal conductivity of Nb-STO polycrystalline ceramics, when the effect of porosity is eliminated.

**Fig. 4:**
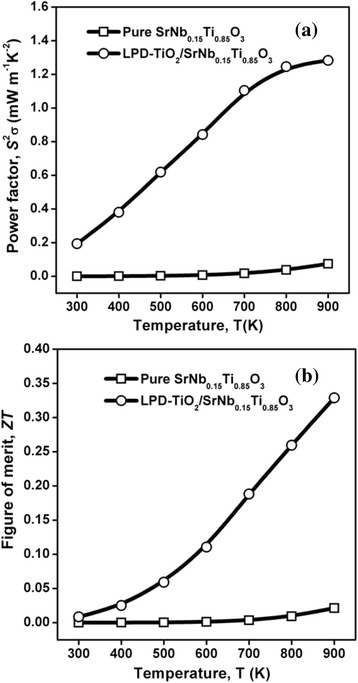
Temperature-dependent thermoelectric power factor *S* ^2^σ (**a**) and dimensionless figure of merit *ZT* (**b**) of Nb-STO composites with and without the surface modification of nanosized titania

The thermoelectric power factor *S*^2^σ and dimensionless figure of merit *ZT* of the sampls are shown in Fig. 4. The power factor of Nb-STO composite with the surface modification of nanosized titania was increased by more than 15 times compared with the pure Nb-STO (Fig. 4a), due to the significant increases of electrical conductivity. The *ZT* value also improved evidently (Fig. 4b), because the enhancement of the power factor was higher than that of thermal conductivity. The highest *ZT* value reached 0.33 at 900 K.

**Fig. 5:**
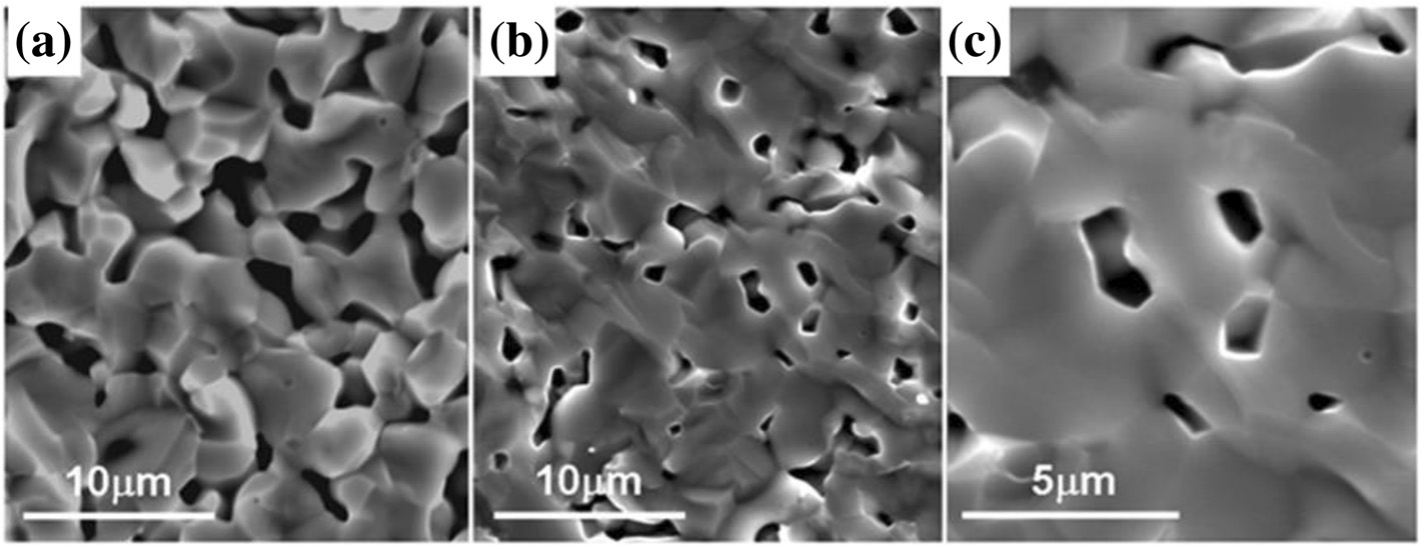
SEM micrographs of Nb-STO composites with (**a**) and without the surface modification of nanosized titania (**b**). **c** Partially enlarged figure of (**b**)

Figure 5 shows the scanning electron micrographs (SEM) of Nb-STO samples with and without the surface modification of nanosized titania. It can be seen that the surface modification of nanosized titania obviously enhanced the grain growth of Nb-STO. When the sintering temperature is higher than 1250 °C, the main sintering mechanism of the SrTiO_3_ ceramic is the volume diffusion [19]. Nb-STO composite with the surface modification of nanosized titania induced Ti-rich composite, which created Sr and O vacancies and therefore enhanced the volume diffusion. Grain growth of Nb-STO lowered the interface scattering of the electrons, promoted carrier mobility [14], and further enhanced the electrical conductivity. It is observed that lots of pores homogeneously distributed in the Nb-STO bulk ceramic (Fig. 5b). The reason for the formation of pores was not clear until now, but these pores homogeneously distributed in the Nb-STO bulk ceramic, which could scatter phonons effectively and reduce the thermal conductivity of Nb-STO composite.

For degenerate semiconductors for which a parabolic band and energy-independent scattering approximation can be assumed [20], the Seebeck coefficient (*S*) can be given by the following equation:

4 $$ S=\frac{8{\pi}^2{k}_B^2}{3e{h}^2}{m}^{*}T{\left(\frac{\pi }{3n}\right)}^{2/3}, $$

where *k*_B_, *h*, m*, and *n* are Boltzmann constant, Planck constant, the effective mass of the carriers, and the carrier concentration, respectively. Equation (4) indicated the Seebeck coefficient mainly depended on carrier concentration. The main reason for the independence of the Seebeck coefficient on the surface modification of nanosized titania is that the carrier concentration not be affected by the surface modification of nanosized titania for it could not react with Nb-STO [10].

## Conclusions

The liquid phase deposition approach was carried out to surface modify the Nb-doped SrTiO_3_ polycrystalline ceramics, and their thermoelectric performances were investigated. The surface modification of nanosized titania enhanced the *ZT* value significantly, because of the increased electrical conductivity, and obtained the highest *ZT* value of 0.33 at 900 K. Enhancement of the electrical conductivity was mainly caused by improved grain growth diminishing the number of the grain boundaries. Newly generated titanium carbide with higher electrical conductivity could also contribute the increased electrical conductivity. Pores homogeneously distributed in the Nb-STO composite could effectively scatter phonons, and hence, contributed to the reduction in the thermal conductivity of Nb-STO composite with the same relative density to pure Nb-STO composite. Seebeck coefficient was almost independent of nanosized titania addition, which was mainly due to the carrier concentration had not been affected by the surface modification of nanosized titania.
